# Supplementary material for: Determinants of early afterdepolarization properties in ventricular myocyte models
Source: PLoS Comput Biol. 2018 Nov 26;14(11):e1006382. doi: 10.1371/journal.pcbi.1006382 (PMC6283611; doi:10.1371/journal.pcbi.1006382)

Supporting Information

We mainly varied the maximum conductance of ionic currents in the AP models to investigate the EAD properties. The control parameter set of each model was set so that it exhibits EADs (Fig.S1).

We added a late I_{Na} (I_{NaL}) to the AP models by changing the h_∞ and j_∞ of the Na channel model as

$$h_\infty = \gamma + (1-\gamma)h_\infty$$
$$j_\infty = \gamma + (1-\gamma)j_\infty$$

where γ determines the portion of incomplete inactivation of the Na channel and thus the magnitude of I_{NaL}. We set the control $\gamma = 0.04$ for all AP models, and varied γ to investigate the effects of I_{NaL} on EAD properties.

We also varied the inactivation time constant (τ_f) of $I_{Ca,L}$ to investigate its effects on EAD properties. The control τ_f is the same as in the original models. In the ORd model, we varied τ_{jCa}.

Only one stimulus was given at t=0 for each parameter set. The initial condition was set by the fully recovered resting state. The stimulus current density and duration were listed for each model

The control parameter sets for each model are listed below. The units of these conductance are the same as in the original models. The parameters listed for each model are the ones we assigned the [0.4p_c, 1.6p_c] for random parameter assignments. The parameters changed from the original models are indicated in **red text** with the original ones in the **parentheses**.

1. **The LR1 model**

   $G_{si} = 0.2\ (\mathbf{0.09}), G_K = 1.0\ (\mathbf{0.282}), G_{K1} = 0.6047, G_{Kp} = 0.0183, G_b = 0.03921.$

   I_{sti}=40μA/cm^2, duration=2 ms.

2. **The LRd model**

   $G_{CaL} = 0.002\ (\mathbf{0.00054}), G_{Ks} = 0.433, G_{Kr} = 0.02614, G_{Ki} = 0.75, G_{Kp} = 0.00552, G_{to} = 0.5, G_{ncx} = 2.5 \times 10^{-4}, G_{nak} = 2.25.$

   I_{sti}= 80 μA/cm^2, duration=0.5 ms.

3. **The HUCLA model**

   $G_{CaL} = 200.0\ (\mathbf{40.0}), G_{Ks} = 0.9\ (\mathbf{0.5}), G_{Kr} = 0.0125, G_{K1} = 0.3, G_{tos} = 0.04, G_{tof} = 0.11, G_{ncx} = 1.0, G_{nak} = 1.5.$

   I_{sti}=40 μA/cm^2, duration = 2 ms.

4. **The TP04 model**

   $G_{CaL} = 8 \times 10^{-4}\ (\mathbf{1.75 \times 10^{-4}}), G_{Ks} = 0.036\ (\mathbf{0.245}), G_{Kr} = 0.01, G_{K1} = 5.405, G_{to} = 0.294, G_{ncx} = 1000.0, G_{nak} = 1.362.$

   I_{sti}= 52μA/cm^2, duration=1ms.

5. **The ORd model**

   $P_{Ca} = 3 \times 10^{-4}\ (\mathbf{1 \times 10^{-4}}), G_{Ks} = 0.008\ (\mathbf{0.0034}), G_{Kr} = 0.0022\ (\mathbf{0.046}), G_{K1} = 0.1908, G_{to} = 0.02, G_{ncx} = 8 \times 10^{-4}, G_{nak} = 30.0.$

   I_{sti}=80 μA/cm^2, duration =0.5 ms.

6. **The GB model**

   $P_{Ca} = 2.875 \times 10^{-4}\ (\mathbf{2.7 \times 10^{-4}}), G_{Ks} = 0.05\ (\mathbf{0.03185}), G_{Kr} = 0.035, G_{Ki} = 0.195, G_{tos} = 0.0113, G_{tof} = 4.2e-4, G_{ncx} = 1.845, G_{nak} = 1.76.$

   I_{sti} = 50 μA/cm^2, duration = 1 ms.

Fig.A. The control action potentials of the AP models.

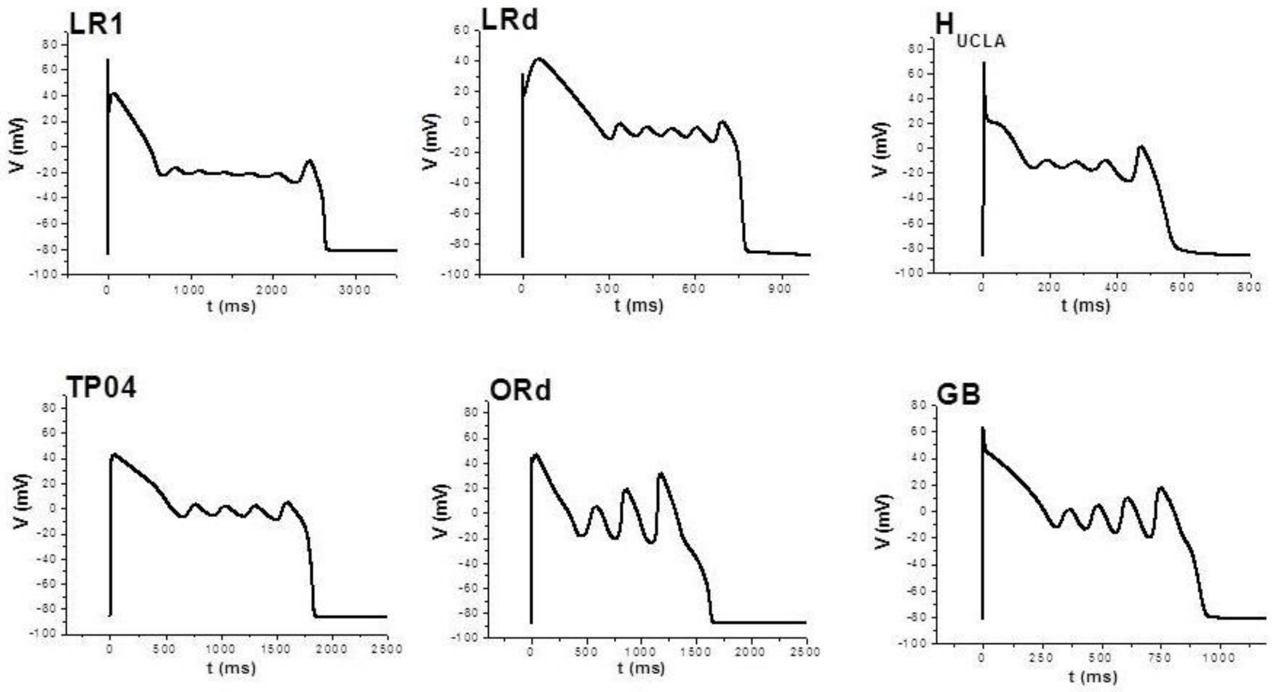

Fig.B. Effects of the maximum conductance of $I_{Ca,L}$ on EAD amplitude in different models.

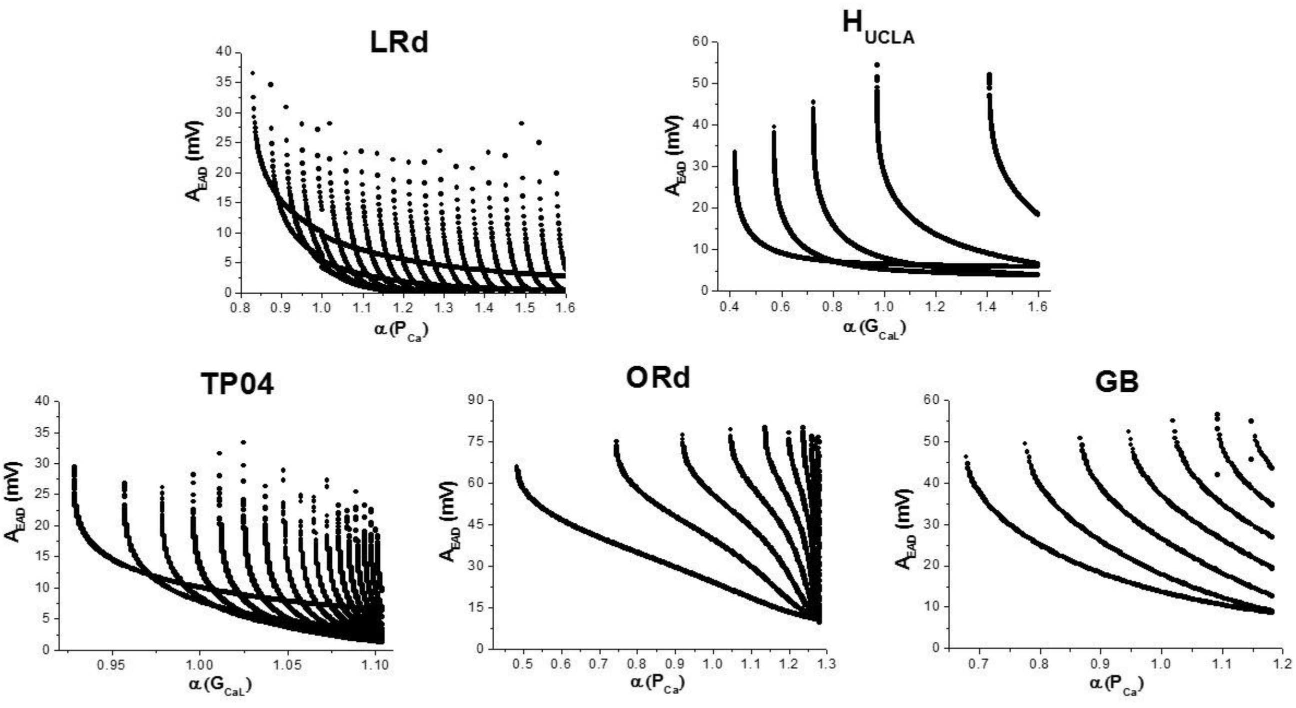

Fig. C. Effects of the maximum conductance of I_{Ks} on EAD amplitude in different models.

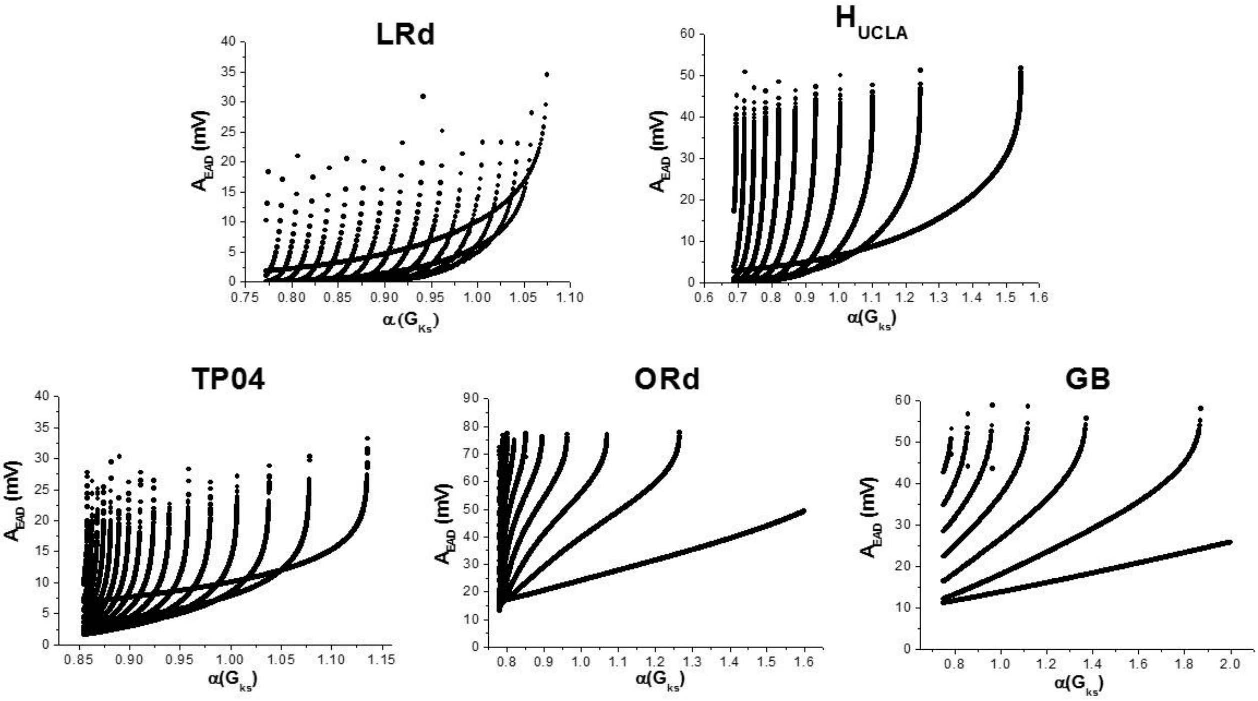

Fig. D. Effects of the maximum conductance of I_{Kr} on EAD amplitude in different models.

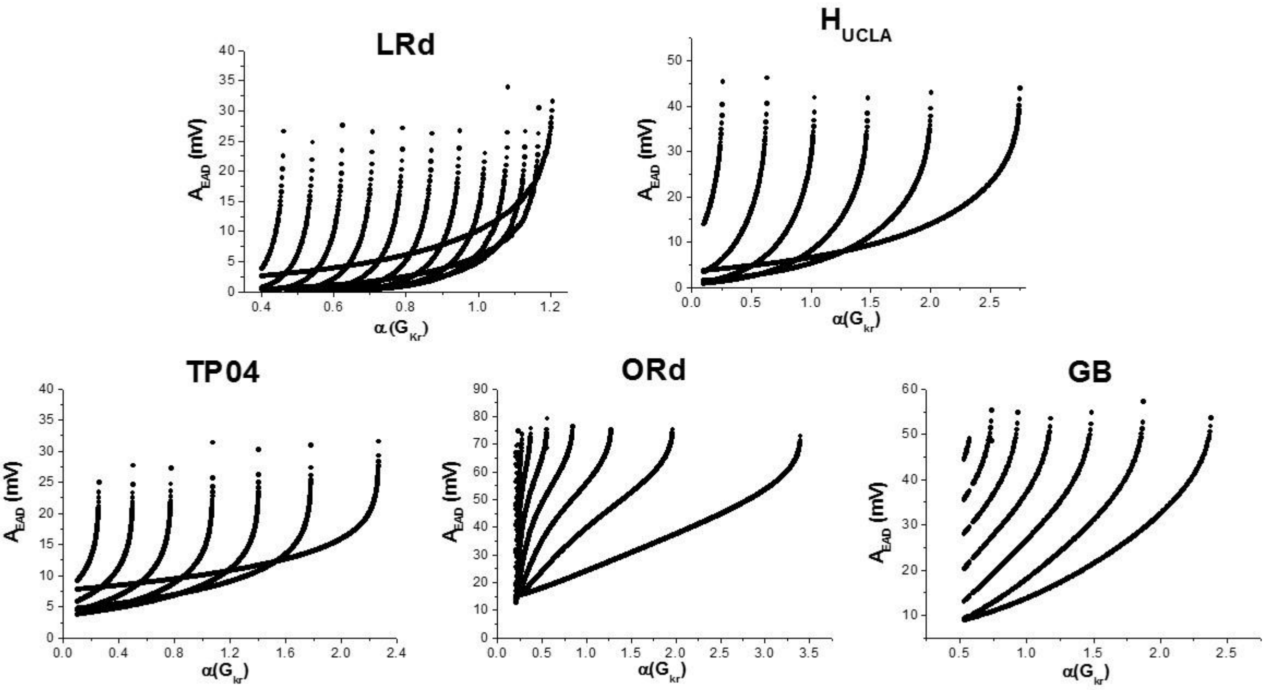

Fig. E. Effects of the maximum conductance of I_{K1} on EAD amplitude in different models.

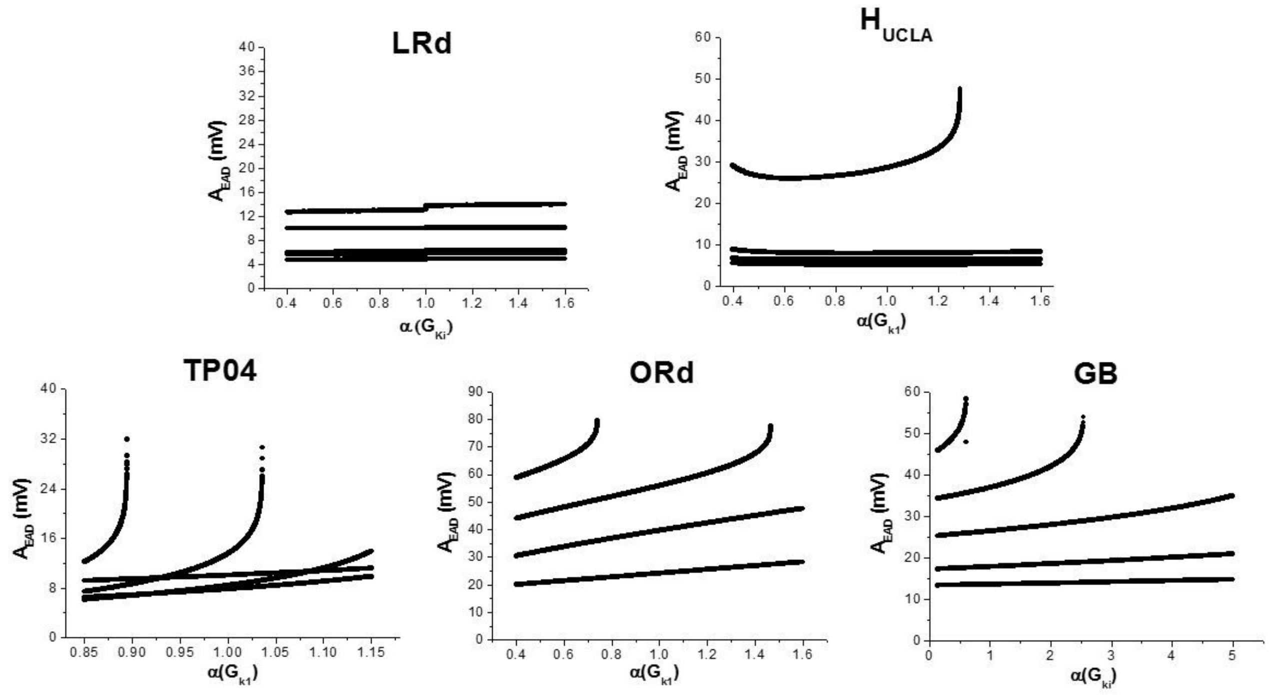

Fig. F. Effects of the maximum conductance of I_{NCX} on EAD amplitude in different models.

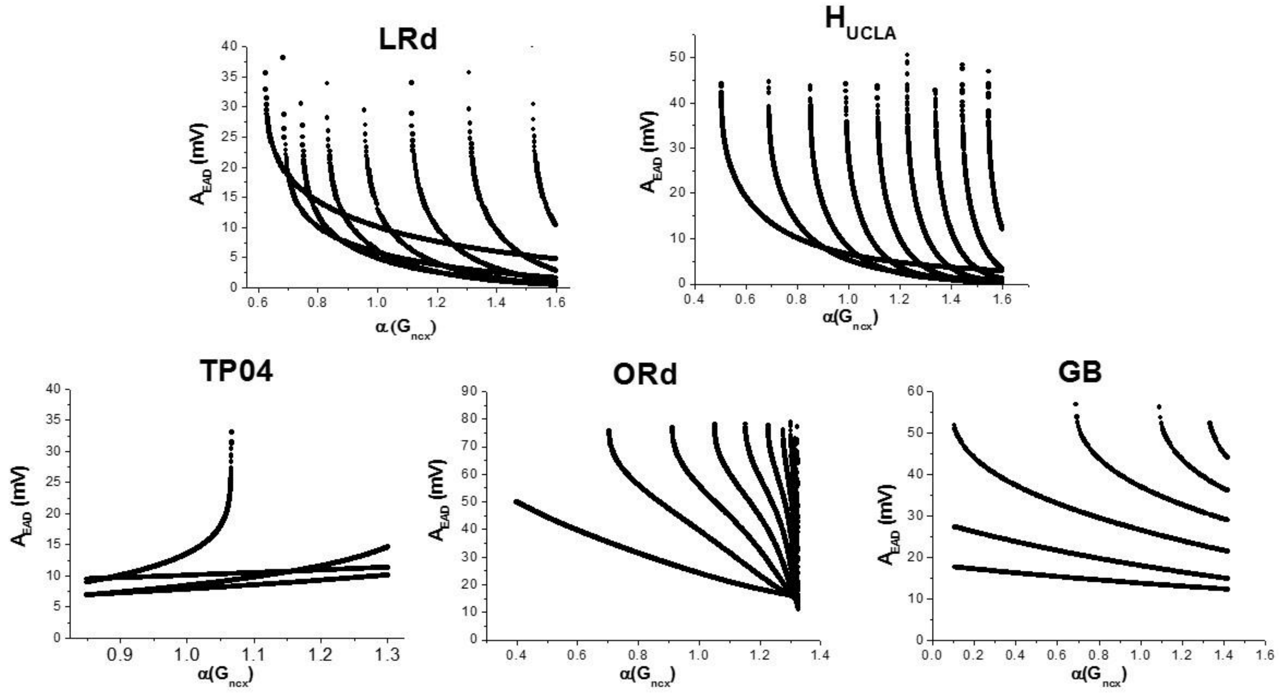

Fig. G. Effects of the maximum conductance of I_{NaK} on EAD amplitude in different models.

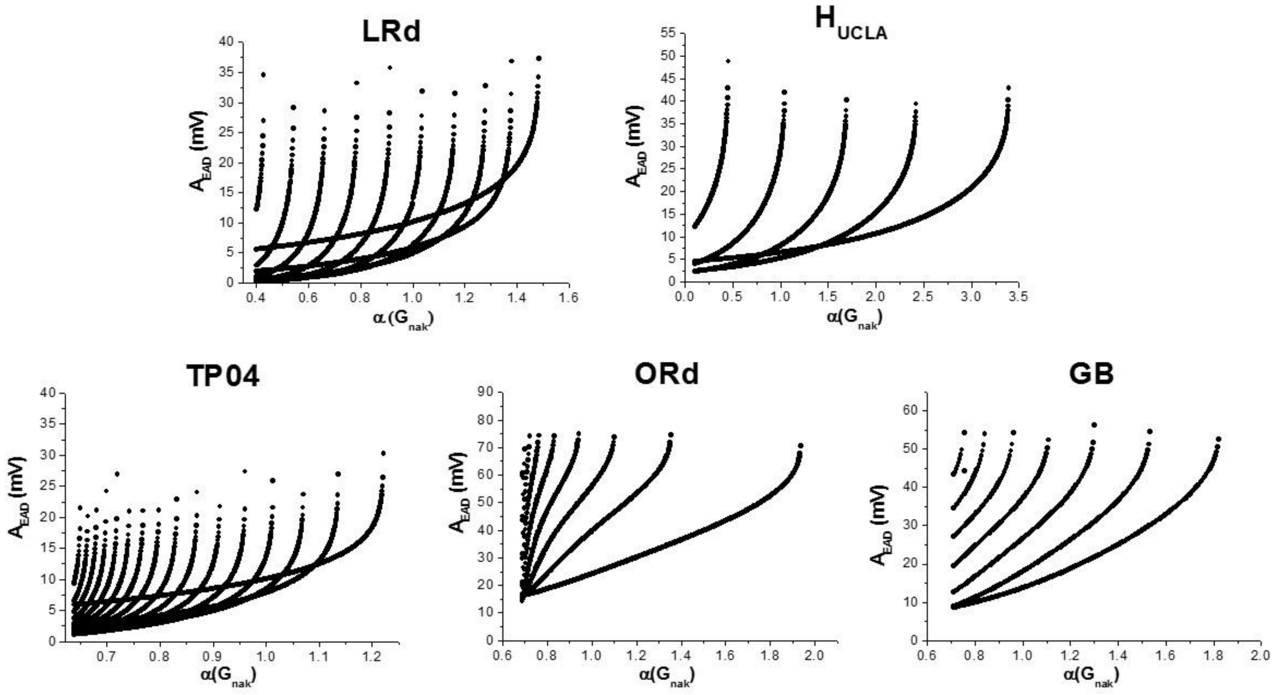

Fig. H. Effects of the maximum conductance of I_{NaL} on EAD amplitude in different models.

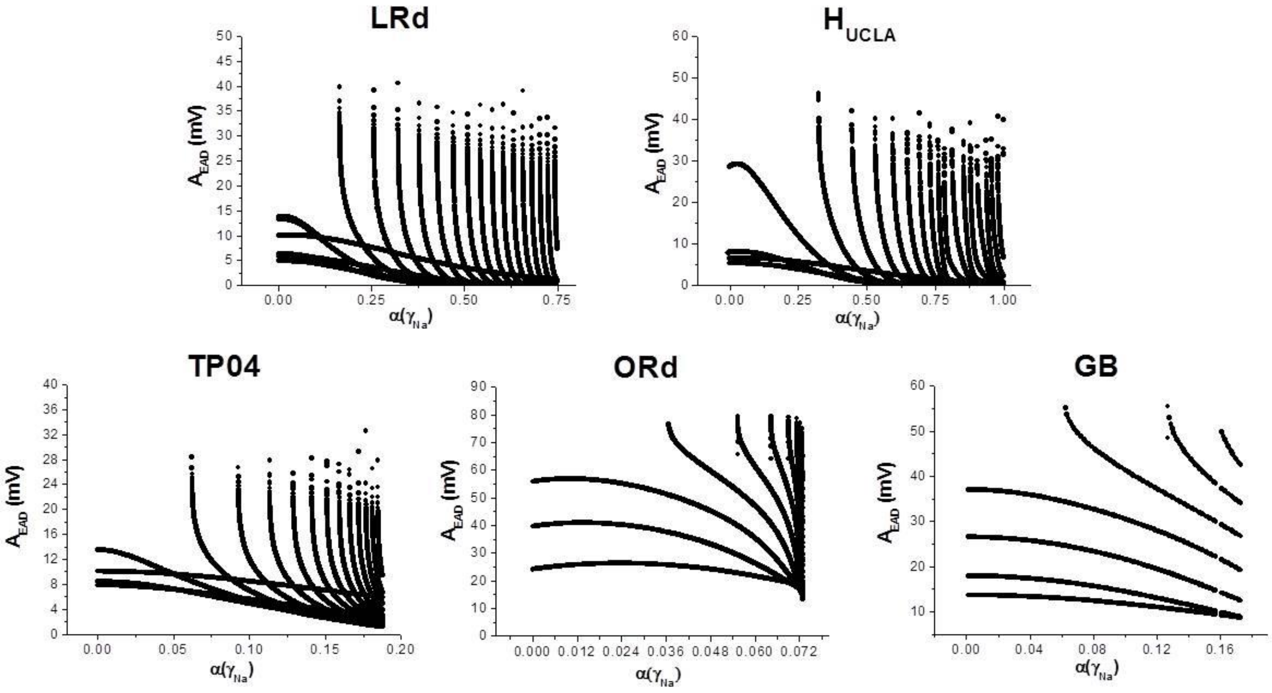

Fig.I. Effects of the maximum conductance of I_{to} on EAD amplitude in different models. Since we used the endocardial ORd and GB models, the control I_{to} conductance is very small, the effects of I_{to} can only be seen when α is very large.

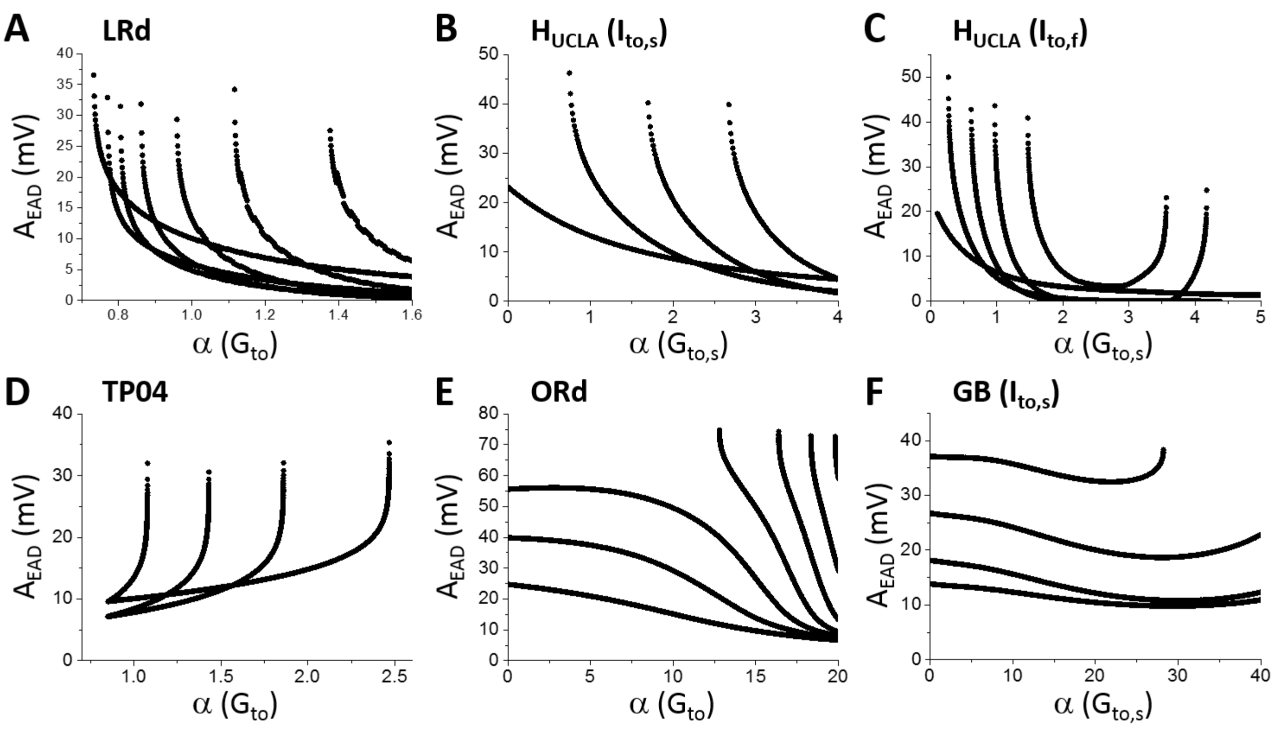

Fig. J. Effects of the inactivation time constant (τ_f) of $I_{Ca,L}$ on EAD amplitude in different models.

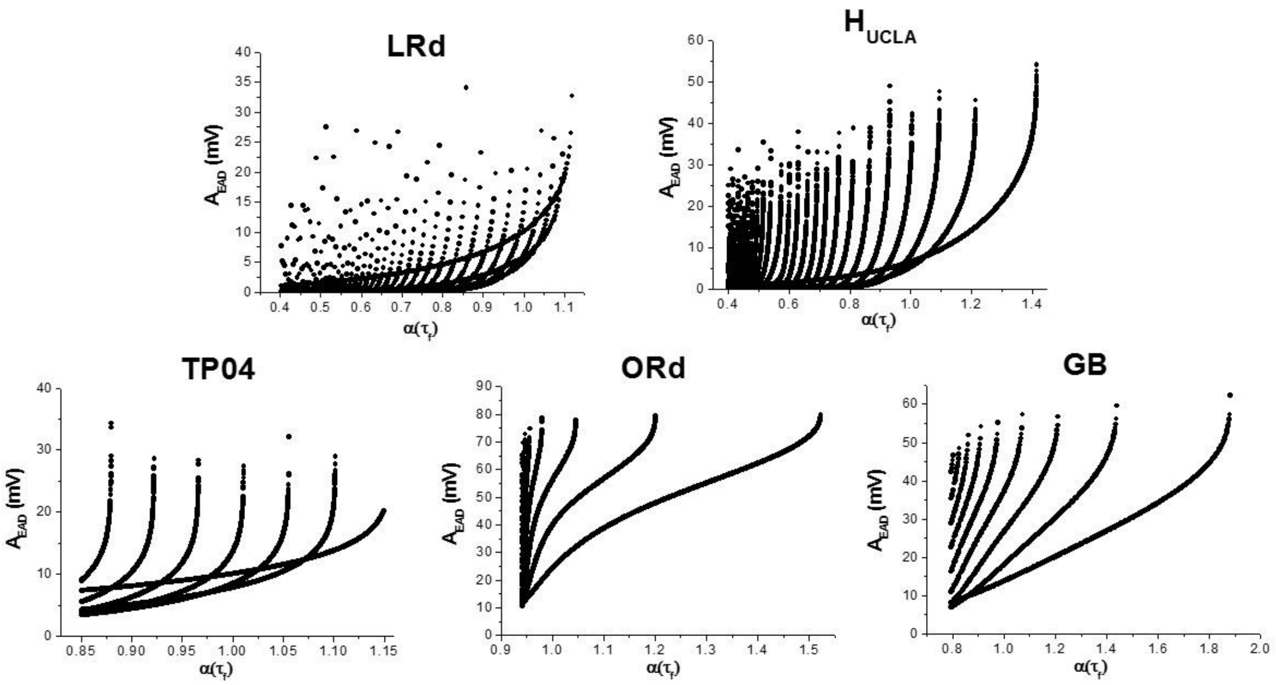

Fig.K. EAD properties of the model by Wilson et al (Chaos, 2017). **A**. Control action potential (black) with EADs. The red trace is the corresponding intracellular Ca^{2+} concentration. The same control parameter set as in the original model was used except 80% reduction of I_{Kr} to induced EADs (G_{Kr} was reduced from 0.045 to 0.009 mS/cm^2). **B**. A_{EAD} versus $V_{takeoff}$ with random parameter sampling in the assigned ranges. The arrow points to the region where a gap of data points occurs. **C**. A_{EAD} versus α (fold change) of the maximum $I_{Ca,L}$ conductance (P_{Ca}). **D**. A_{EAD} versus α (fold change) of the maximum I_{Ks} conductance (G_{Ks}). **E**. T_{EAD} versus α (fold change) of the maximum conductance of different ionic currents as indicated by the colored symbols. **F**. T_{EAD} histogram. The arrow points to the abrupt change in T_{EAD} counts. **G**. L_{EAD} versus α (fold change) of the maximum conductance of difference ionic currents as indicated by the colored symbols. **H**. L_{EAD} histogram. We thank Drs. Wilson and Salama for providing us the original Matlab code of the model.

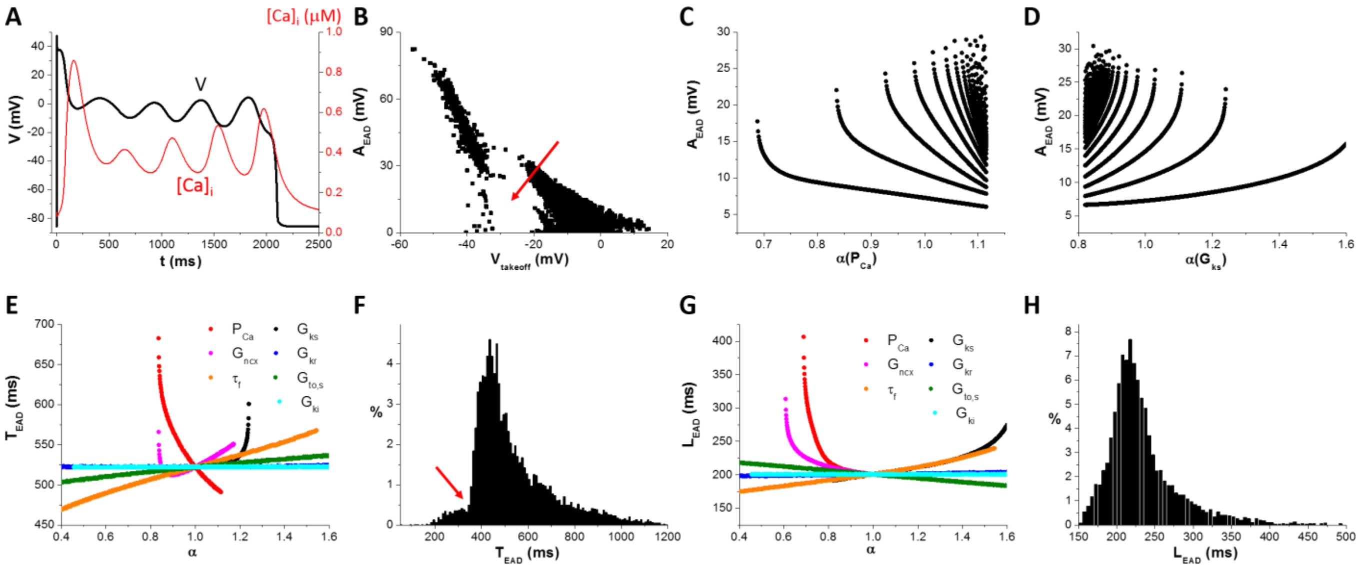

Supplement: S1 Text — The online supporting information includes: 1) The control parameters of each AP model and their changes from the original model to promote EADs; 2) The formualation of INaL; and 3) The supplemental results shown as supplemental figures, from Fig A to Fig K. (PDF) [file pcbi.1006382.s001.pdf]
